# Irisin: a new molecular marker and target in metabolic disorder

**DOI:** 10.1186/1476-511X-14-2

**Published:** 2015-01-14

**Authors:** Jia-qi Chen, Yue-ye Huang, Aaron M Gusdon, Shen Qu

**Affiliations:** Department of Endocrinology and Metabolism, Shanghai 10th People’s Hospital, School of Medicine, Tongji University, Shanghai, 200072 China; Nanjing Medical University, Nanjing, Jiangsu 210029 China; Department of Neurology, Weill Cornell Medical College, New York, NY 10065 USA

**Keywords:** Irisin, Obesity, Metabolic diseases

## Abstract

Irisin is a newly discovered exercise-mediated myokine which regulates energy metabolism and has been the subject of much recent research. Irisin plays an important role in metabolic diseases making it a potential new target to combat obesity and its associated disorders, such as T2DM. However, the results of several recent studies investigating the effects of irisin have been controversial. The present review will introduce the discovery of irisin, the role of irisin in metabolic disorders, possible mechanisms, and unanswered questions for future research.

## Introduction

Irisin is a newly discovered exercise-mediated myokine which regulates energy metabolism by inducing browning of white adipose tissue and thus dissipates chemical energy in the form of heat. Bostrom et al. [1] revealed that exercise stimulates PPAR-γ co-activator-1α (PGC-1α), which in turn upregulates its downstream target fibronectin type III domain containing 5 (FNDC5), thereafter the C-terminus is cleaved and the remaining 112 amino acid peptide is referred to as irisin. By increasing the mRNA expression of UCP1 and Cidea, irisin causes browning of primarily subcutaneous and visceral adipose tissue and thereby induces thermogenesis. However, Wu et al. [2] reported that only part of the subcutaneous adipose tissue inverts into brown adipose tissue (BAT), with a substantial portion not demonstrating features consistent with classical white adipose tissue (WAT) or BAT which they refer to it beige adipose tissues.

Irisin has gained great interest as a potential new target to combat obesity and its associated disorders, such as type 2 diabetes mellitus (T2DM). For Bostrom et al. [1] reported that moderate increases in circulating irisin levels augments energy expenditure, protects against diet-induced weight gain and alleviates insulin resistance. Numerous studies focus on the association of irisin with metabolic disease. This review discusses the role of irisin in obesity, diabetes, and other metabolic diseases.

## Irisin in obesity and metabolic syndrome

Irisin has initially been described as a protective factor against diet-induced weight gain, mediated by browning of WAT and thus increased energy expenditure [1]. Many subsequent studies have investigated the association of circulating irisin with obesity in humans (summarized in Table 1).

**Table 1:** **The association between irisin levels and obesity**

	Population	Group size	Study design
Positive correlation between irisin levels and obesity			
Huh et al. [3]	Healthy middle-aged women with wide range of BMI	117	Cross-sectional
Stengel et al. [4]	Anorexia nervosa patients, normal weight control subjects, and morbidly obese patients	40	Cross-sectional
Pardo et al. [5]	Anorexia nervosa patients, normal weight control subjects, and morbidly obese patients	145	Cross-sectional
Liu et al. [6]	Diabetic subjects and non-diabetic subjects	156	Cross-sectional
Huh et al. [3]	Subjects undergoing gastric banding or gastric bypass surgery	14	Interventional
De la Iglesia et al. [13]	Subjects following an 8-week hypocaloric dietary strategy	93	Interventional
Crujeiras et al. [8]	Obese patients undergoing a weight loss program consisting of 8 weeks of a hypocaloric diet and follow-up at 16-weeks	94	Interventional
Negative correlation between irisin levels and obesity			
Moreno-Navarrete et al. [10]	Non-diabetic subjects	69	Cross-sectional
Choi et al. [11]	NGT subjects and new-onset T2DM subjects	208	Cross-sectional

Several studies have investigated the relationship between circulating irisin and body mass index (BMI). Huh et al. [3] reported in a cross-sectional study of 117 healthy middle-aged women with BMI ranging from 20.0 to 47.7 kg/m2, circulating irisin had positive associations with fat-free mass and a positive trend with BMI. Stengel et al. [4] performed a study including anorexia nervosa patients, normal weight control subjects, and morbidly obese patients, providing a broad spectrum of body weights. They found that obese patients have higher circulating irisin levels compared with normal weight controls and anorexic patients, and irisin has a positive correlation with body weight and BMI. Similarly, Pardo et al. [5] reported that in 145 female patients, including anorexia nervosa patients, obese patients, and healthy normal weight subjects, the plasma irisin levels are significantly elevated in the obese patients compared with the anorexia nervosa patients and normal weight subjects, and irisin also positively correlated with body weight, BMI, and fat mass. Liu et al. [6] found that in non-diabetic subjects, circulating irisin is correlated with BMI. Their group also reported that in patients with T2DM and renal insufficiency, irisin levels correlated with BMI, fat mass, and percentage of fat mass [7]. Additional studies (Crujeiras et al. [8] & Park et al. [9]) have also shown that plasma irisin levels are positively correlated with weight, BMI, waist circumference, and fat mass.

However, the results from other studies have been controversial. Moreno-Navarrete et al. [10] studied a subgroup of 69 non-diabetic subjects with BMI 27.61 ± 3.8, and reported that circulating irisin decreased in association with obesity. In fact, they showed that circulating irisin was negatively associated with BMI, percent fat mass, and waist to hip ratio. Choi et al. [11] found that plasma irisin was negatively correlated with BMI in a study population including 104 subjects with normal glucose tolerance and 104 subjects with new-onset T2D. Sanchis-Gomar et al. [12] did not find a positive or negative correlation between circulating irisin levels and BMI.

The discrepancies in the above mentioned studies may be due to different populations analyzed in the studies, as some included obese subjects with metabolic diseases, which may influence plasma irisin levels.

Studies looking at patients undergoing interventions provide further evidence supporting the positive correlation between BMI and circulating irisin levels. Blood samples were collected at baseline and 6 months after surgery in a subgroup of 14 obese subjects who were undergoing gastric banding or gastric bypass surgery [3]. Circulating irisin levels decreased significantly after 6 months, which was accompanied by significant weight loss. De la Iglesia et al. [13] studied ninety-three Caucasian adults diagnosed with metabolic syndrome, who followed an 8-week hypocaloric dietary strategy. They found plasma irisin levels were significantly reduced at the end of the study accompanying the weight loss. Crujeiras et al. [8] reported in a group of 94 obese patients (BMI 35.66 ± 4.5 kg/m2), after a weight loss program consisting of 8 weeks of a hypocaloric diet and follow-up at 16-weeks. Irisin levels decreased paralleling body weight reduction after caloric restriction at 8 weeks, and returned to baseline levels at 24 weeks in those patients who regained the lost weight.

Additionally, a lower fat mass may also have contributied to the lower irisin levels. Roca-Rivada et al. [14] found secretion of irisin was higher from white adipose tissues of diet-induced obese rats compared to lean controls, suggesting that adipose tissue, especially in obesity, was an important source of irisin. Consistent with this view, some studies also found circulating irisin was positively correlated with fat mass in human [4, 5, 8–10, 15, 16].

In summary, despite some controversy, it is generally believed that there is a positive correlation of circulating irisin with obesity, which is in apparent conflict with the proposed anti-obesity effect of irisin. A possible explanation which could reconcile the controversial data may be that irisin acts as a physiological protective factor against obesity mediated by the browning of WAT and is thus increased in compensation for increasing body mass. In pathological states of morbidity obesity, physiological irisin cannot maintain the balance of energy storage and expenditure, and additional irisin is secreted from skeletal muscle, and even adipose tissue can produce irisin as compensation for dramatically increased fat storage.

Metabolic syndrome (MetS) is associated with a number of metabolic disorders and may be caused by obesity, especially central obesity.

Hee et al. [16] reported that baseline irisin levels are significantly higher in subjects with MetS than in subjects without MetS, while Yan et al. [17] reported that circulating irisin levels are significantly reduced in subjects with MetS than controls. This may be due to the different population recruited in these studies, as the study from Hee included subjects with MetS with high BMI making it the most important factor while the study from Yan included subjects with central obesity making glucose homeostasis and insulin resistance (IR) the major factors (discussed below).

## Irisin in DM and glucose homeostasis

A moderate increase in circulating irisin levels augmented energy expenditure, not only reducing weight gain due to a high-fat diet, but also improving diet induced IR [1]. As irisin is a novel myokine secreted in response to PGC-1α activation. Studies suggest that PGC-1α is important for mitochondrial homeostasis for it regulates mitochondrial biogenesis and oxidative metabolism, and mitochondrial function also plays a role in IR. Furthermore, expression and activity of PGC1-α are lower in patients with T2DM [18–20].

Choi et al. [11] compared serum irisin levels in new-onset T2DM patients with normal glucose tolerance (NGT) controls, and found that serum irisin levels are significantly decreased in the new-onset T2DM group. Moreover, increased irisin levels are associated with reduced odds of newly diagnosed T2DM. Xiang et al. [21] reported that in newly diagnosed T2DM subjects, serum irisin concentrations are significantly lower than that in controls. Kurdiova et al. [22] also reported similar results in drug-naive T2DM subjects. This phenomenon is also observed in long-term T2DM patients which has been reported in several other studies [6, 10, 23].

Yuksel et al. [24] performed a case–control study of 20 women with gestational diabetes mellitus (GDM) and 20 pregnant women without GDM. Maternal serum irisin levels were significantly lower at the time of birth in women with GDM compared to controls, while cord blood irisin levels are not significantly different. Kuzmicki et al. [25] also found that serum irisin concentration was significantly lower in patients with GDM compared with NGT subjects; but the significant difference between the two groups disappeared 3 months after delivery. However two other studies reported conflicting results. Ebert et al. [26] found that in GDM patients, circulating irisin levels were significantly higher than that in healthy, pregnant, gestational age-matched controls after delivery, but did not differ during pregnancy. Piya et al. [27] studied pregnant women undergoing elective caesarean-section, and reported that in obese and GDM subjects, fasting serum irisin before caesarean section was significantly higher compared with controls, after adjusting for BMI, lipids, and blood glucose.

In addition to the above findings, the association between irisin and glucose homeostasis has also reported. These results have also been controversial. Choi et al. [11] found that serum irisin was significantly negatively correlated with 2 h plasma glucose and hemoglobin A1c (HbA1c). Furthermore, multiple regression analysis showed that 2 h plasma glucose was an independent variable influencing serum irisin levels. Yan et al. [17] also reported in obese Chinese adults, serum irisin was significantly negatively associated with fasting insulin and HbA1c. On the other hand, Liu et al. [6] found that circulating irisin was significantly positively correlated with fasting blood glucose. Hee Park et al. [16] also reported that circulating irisin was associated positively with fasting glucose and homeostasis model assessment of insulin resistance (HOMA-IR). Sesti et al. [28] found that irisin levels were positively correlated with fasting and 2 h post-load insulin levels, and were negatively correlated with insulin-stimulated glucose disposal, and insulin clearance. However, Stengal et al. [4] did not find an association between serum irisin and blood glucose levels, but found that serum irisin was positively correlated with insulin levels.

Crujeiras et al. [29] reported in a study of 136 obese patients who followed an eight-week hypocaloric diet that after a successful dietary intervention, 50% of the patients who regained the lost weight during the follow-up period were categorized as insulin resistant (HOMA-IR ≥ 2.5) compared with only 25% of patients who maintain the weight loss. And increased risk of IR during the follow-up period was related to high irisin levels at baseline.

Al-Daghri et al. [30] conducted a study in a cohort of 153 Saudi Arabian children, and found that girls have higher circulating irisin levels than boys. There was a significant correlation between circulating irisin and fasting blood glucose. And in girls, but not in boys, HOMA-IR correlated negatively with irisin levels.

In addition, serum irisin was also associated with glucose homeostasis in pregnancy. Kuzmicki et al. [25] reported in pregnant patients, irisin concentration was negatively associated with 2 h glucose levels. In the women with NGT, irisin concentration was negatively associated with 2 h insulin level and HOMA-IR. This phenomenon was also reported by Yuksel et al. [24] that serum irisin level is negatively correlated with HOMA-IR in pregnancy. However, Ebert et al. [26] found that fasting insulin was positively associated with serum irisin during pregnancy.

In conclusion, circulating irisin levels are lower in both newly-diagnosed and long-term T2DM, which indicates that irisin can be a predictor of T2DM (Table 2). The decrease in irisin may due to lower PGC-1α expression and activity. However for GDM, results were not consistent, this may due to the fact that irisin was detected at different times during pregnancy or after delivery. The association between irisin and glucose homeostasis was confirmed by several studies, indicating that irisin may be a predictor and protective factor for developing diabetes. However, whether it is negatively or positively concerned, still remains controversial. Different populations studied may partially explain the different results. For Choi et al. [11] and Yan et al. [17], the populations included individuals with little or no IR. However for Liu et al. [6] and Hee Park et al. [16], the populations may have had more severe IR and irisin was increased in response to the deterioration of insulin sensitivity.

**Table 2:** **Level of irisin in other pathological condistions**

Pathological conditions	Level of irisin	Reference
Newly diagnosed T2DM	Decrease	Choi et al. [11] Xiang et al. [21] Kurdiova et al. [22]
Long-term T2DM	Decrease	Liu et al. [6] Moreno-Navarrete et al. [10] Zhang et al. [23]
GDM	Decrease	Yuksel et al. [24] Kuzmicki et al. [25]
	No difference	Ebert et al. [26]
MetS	Increase	Hee et al. [16]
	Decrease	Yan et al. [17]
NAFLD	Decrease	Zhang et al. [34] Polyzos et al. [35]
	Increase	Choi et al. [36]
PCOS	Increase	Chang et al. [37]
CKD	Decrease	Liu et al. [7] Wen et al. [38] Ebert et al. [39]

## Irisin in other metabolic disorders

Besides obesity and T2DM, many studies assessed the role of irisin in other metabolic diseases (Table 2).

Earlier studies have shown that non-alcoholic fatty liver disease (NAFLD) and polycystic ovary syndrome (PCOS) are associated with MetS and its components [31, 32]. De la Iglesia et al. [13] reported that in MetS subjects, the depletion of irisin after a weight loss program significantly correlated with changes in total cholesterol, total cholesterol/high-density lipoprotein (HDL) cholesterol ratio, low-density lipoprotein (LDL) cholesterol and apolipoprotein B, independently of changes in body weight. Panagiotou et al. [33] reported that in subjects with high cardiovascular risk, irisin was negatively associated with HDL cholesterol and large HDL particles after adjusting for age, sex, and race.

Zhang et al. [34] found that serum irisin levels were reduced in obese adults with NAFLD. Serum irisin levels were reduced gradually with the increase of intrahepatic triglyceride content. Higher serum irisin levels were associated with preferable triglyceride levels. Additionally serum irisin levels were independently associated with liver fat. Polyzos et al. [35] also found that serum irisin levels were significantly decreased in patients with NAFLD and non-alcoholic steatohepatitis (NASH) compared with lean controls, but do not differ between patients with NAFLD and NASH. This difference remains significant after adjustment for BMI or waist circumference, gender, age, and IR. However, Choi et al. [36] found that in 355 health screen examinees, the subjects with NAFLD had significantly higher irisin levels than the subjects without NAFLD. These results suggest that irisin may play an important role in lipid metabolism and thus in the development of NALFD, and the mechanism behind it needs further researches.

Chang et al. [37] reported that serum irisin levels in PCOS patients are significantly elevated as compared to those of controls. Remarkably, levels of fasting irisin remained significantly elevated in PCOS without MetS risk factors and healthy-weight PCOS patients when compared to matched controls. This indicates that irisin might be an independent factor that contributes to the development of PCOS and may also represent a novel PCOS biomarker.

Liu et al. [7] reported circulating irisin was significantly decreased in T2DM with renal insufficiency and the reduction in irisin was most pronounced in stage 5 chronic kidney disease (CKD) patients. Wen et al. [38] found that irisin levels were significantly decreased in patients with stage 5 CKD than in age- and sex-matched normal subjects. The decrease in irisin levels was inversely correlated with the levels of blood urea nitrogen and creatinine. Ebert et al. [39] studied a patient population with stage 1 to 5 CKD, and found that serum irisin levels significantly decreased with increasing CKD stage and are lowest in patients with stage 5 CKD. These results suggest that irisin is associated with renal function and that irisin may not be renally excreted. The possible mechanism may involve negative feedback on myocytes by increasing serum creatinine resulting in decreased irisin secretion.

In addition, Anastasilakis et al. [40] studied irisin levels in postmenopausal women with low bone mass and age-matched controls. They found that serum irisin levels were not significantly different between women with or without low bone mass and are not affected by either denosumab or teriparatide treatment for 3 months. However irisin levels were lower in women with than without previous osteoporotic fractures, and were negatively associated parathyroid hormone. Singhal et al. [41] reported irisin levels were positively associated with bone density Z-scores, volumetric bone mineral density (total and trabecular), stiffness, and failure load in 85 adolescent women. These results did not reveal the association between irisin and bone metabolism clearly, and further researches are needed.

## Summary

The discovery of irisin and its role in metabolic diseases may reveal a new target for obesity and its related diseases, like T2DM, and NAFLD, although different research groups have reported conflicting results. There are still many questions to be answered. Most importantly, the mechanism of action of irisin in metabolic diseases remains incompletely understood. Only once the underlying mechanisms have been revealed, can we explain the discrepancies between reported results, and understand the true role that irisin plays in a variety of metabolic diseases. Other gaps to be filled in further studies include identification of the irisin receptor, the molecular pathways regulating irisin, and other functions of irisin.

## Abbreviations

PGC-1α: PPAR-γ co-activator-1α
FNDC5: Fibronectin type III domain containing 5
BAT: Brown adipose tissue
WAT: White adipose tissue
T2DM: Type 2 diabetes mellitus
BMI: Body mass index
MetS: Metabolic syndrome
IR: Insulin resistance
NGT: Normal glucose tolerance
GDM: Gestational diabetes mellitus
HbA1c: Hemoglobin A1c
HOMA-IR: Homeostasis model assessment of insulin resistance
NAFLD: Non-alcoholic fatty liver disease
PCOS: Polycystic ovary syndrome
HDL: High-density lipoprotein
LDL: Low-density lipoprotein
NASH: Non-alcoholic steatohepatitis
CKD: Chronic kidney disease.
